# Spontaneous Multiloculated Lumbar Abscess in a Middle-Aged Male With Unexplained Progressive Back Pain and Muscle Weakness

**DOI:** 10.7759/cureus.27346

**Published:** 2022-07-27

**Authors:** Mary Ann Nyc, La'Kesha Francis, Jason R Woloski

**Affiliations:** 1 Family Medicine, University of California, Riverside, Riverside, USA; 2 Family Medicine, Geisinger Health System, Wilkes-Barre, USA; 3 Family Medicine, Geisinger Health System, Geisinger Commonwealth School of Medicine, Wilkes-Barre, USA

**Keywords:** lower extremity weakness, atypical back pain, back pain, treatment of spinal epidural abscess, spinal epidural abscess

## Abstract

A 54-year-old man with a past medical history significant for sciatica, as well as multiple orthopedic surgeries with hardware, was transferred from an outside rural facility for further workup of a two-month history of progressive back pain and muscle weakness. Investigations ultimately revealed abnormal enhancement from T11 to sacrum, with a large epidural abscess from L5 to the sacrum, best visualized on an MRI. Following the MRI confirmation of loculated complex thoracolumbar abscess, neurosurgery performed a left L3-S1 unilateral laminotomy and evacuation of compressive multiloculated epidural abscesses. The patient was then treated with empirical antimicrobial coverage for epidural abscess with vancomycin and ceftriaxone, which was narrowed to cefazolin based on positive methicillin-susceptible *Staphylococcus aureus* (MSSA) wound cultures obtained in the operating room. The patient completed a total six-week course of antibiotic therapy. Apart from some superficial wound dehiscence postoperative, the patient ultimately recovered well and had a resolution of most presenting symptoms.

## Introduction

An epidural abscess is a rare but serious pyogenic infection of the central nervous system. There are two types of epidural abscesses: spinal epidural abscesses (SEA) and intracranial epidural abscesses (IEA). When the infection spreads within the enclosed bony space of the spinal column, as in a SEA, it can compress the spinal cord and lead to severe symptoms, complications, and fatality. The challenge is that SEA can manifest in different ways, often with initially nondescript and vague symptoms such as back pain, numbness, tingling, weakness, and, in some cases, abdominal pain [1]. Therefore, it is crucial to diagnose SEA in time to avoid irreversible damage.

Furthermore, timely treatment is necessary for patient safety, prevention of irreversible sequelae, and promoting favorable outcomes in medical malpractice lawsuits that commonly follow such cases [2]. Though the incidence rate for SEA is relatively low at 1.2 cases per 10,000 per year, the rate has been increasing since the 1980s. This is thought to be partly due to the aging population, improved imaging techniques for diagnosis, prevalent intravenous drug abuse, immunocompromised states, and more common use of spinal surgical procedures and injections [3-5].

SEA most commonly affects elderly patients in their 60s and 70s. Risk factors include immunodeficiency (diabetes, cancer, alcoholism, cirrhosis, end-stage renal disease, and HIV/AIDS), epidural procedures (anesthesia, steroid injections, epidural catheter placement, and lumbar puncture), bacteremia, and spinal surgery. Among these, diabetes accounts for the greatest risk factor [4,5].

In this article, we describe a case of SEA that presented with a combination of symptoms, risk factors, and exposures that expanded the differential and prompted questions about the source of infection.

## Case presentation

A 54-year-old man with a past medical history significant for sciatica, as well as multiple orthopedic surgeries requiring titanium and stainless steel hardware, and recent pneumonia treated with antibiotics, was transferred from an outside rural facility for further workup of a two-month history of progressive back pain and muscle weakness, advanced imaging, and potential neurosurgery consultation. History was obtained from the patient and his wife. The onset of symptoms seemed to have coincided with a possible mechanical fall while at work, where he was able to stop his fall with his hands. On the same day of this incident, the patient was also experiencing pain in his lower left back, with associated vomiting, and was subsequently treated for a suspected “kidney infection.” Since then, the patient’s back pain progressed to the bilateral lower back areas as well as the sacral area. The pain was described as distinctly unlike his usual “sciatic pain.” It was reported to be sharp in nature, persistent, and only improved when he was either supine or laying on his sides. When asked to rank the pain in severity from zero to 10, with 10 being the most severe, the patient quantified it as 4/10. The patient endorsed chills but no fever. He also noticed lower extremity weakness. The weakness was associated with limb shaking when the patient stood and was relieved once he started walking.

The patient also reported an unintentional weight loss of approximately 60 pounds over a total of eight months, with 20 of those pounds lost in the past month prior to admission. However, he mentioned significantly reduced oral intake as a result of the symptoms, which likely contributed to weight loss. The patient was also diagnosed with pneumonia three weeks prior to admission. He was unsure which antibiotics he received for that infection. Furthermore, he reported one episode of urinary incontinence a few days prior to admission. A review of systems also uncovered constipation, with the patient’s last reported bowel movement seven days prior to admission.

The patient indicated recent episodes of expressive aphasia. He denied chest pain, saddle anesthesia, fecal incontinence, loss of consciousness, syncope, seizures, recent falls, tick bites, or recent travel. The patient lived with his family in a heavily wooded area of Northeast Pennsylvania and was employed at a job involving lake and water purification.

On general examination, the patient was normotensive with a blood pressure of 134/84, temperature of 37.6°C (99.7°F), heart rate of 108 bpm, and respiratory rate of 18. The musculoskeletal exam revealed sacral bony tenderness to palpation without erythema or step-offs. Left lower back was also tender to palpation at myofascial points. The skin exam showed cool extremities. Neurological exam exhibited mild slowness of speech, hyporeflexia of lower extremities, 3/5 strength in his lower extremities, and a positive straight leg test on his left side. No fasciculations or muscle atrophy was noted in all the extremities. Gait appeared ataxic, and the patient was unable to tandem walk. However, a full assessment of the gait was difficult due to the patient’s discomfort with ambulation.

Investigations

A non-contrast CT of the head was ordered, with unremarkable findings. A multiview lumbar spine x-ray was ordered to further investigate the cause of the patient’s back pain. The radiology report described degenerative changes, including loss of disc space height, particularly at L5-S1, osteophyte formation, and lower lumbar facet arthropathy.

**Figure 1:**
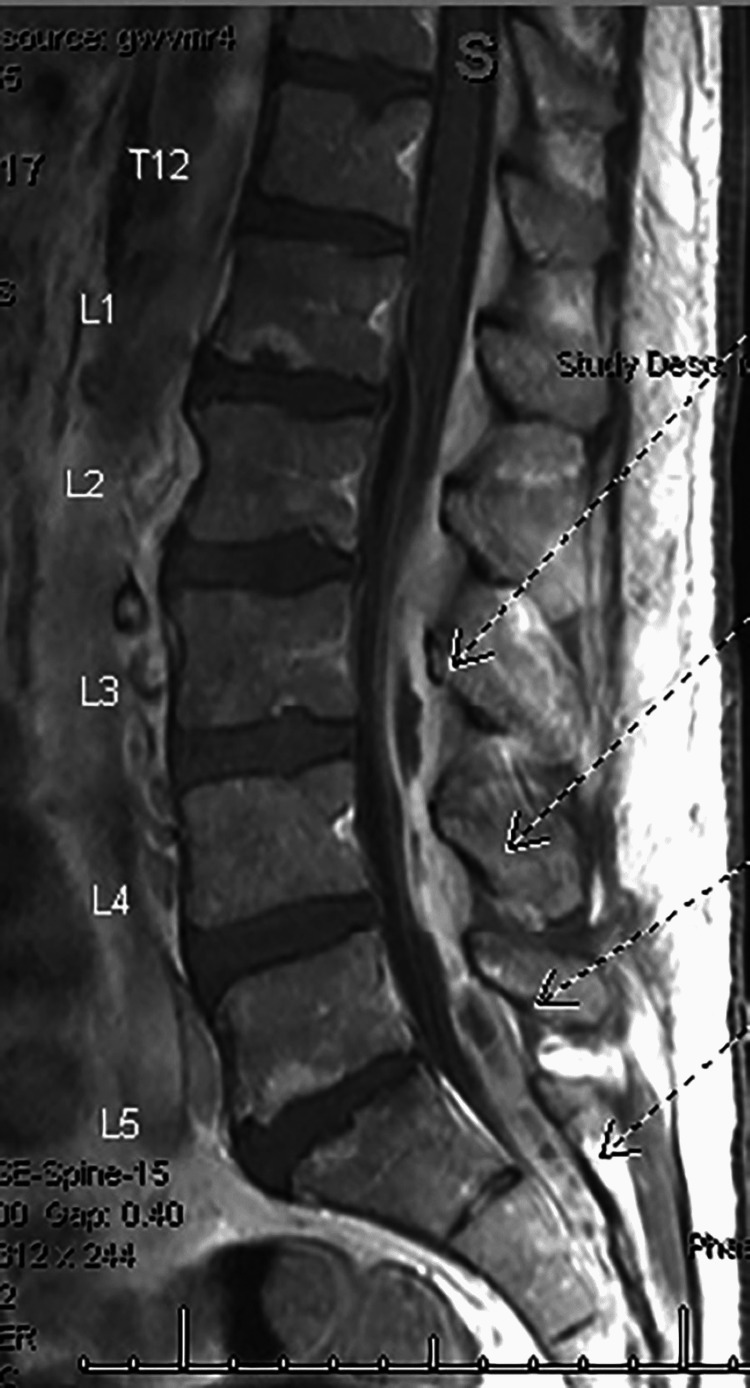
MRI (SAG T1 + contrast sequence) showing a long segment of epidural enhancement compatible with epidural phlegmon/abscess, extending T12 through the imaged sacral levels, which contributes to a varying degree of the spinal canal and neural foraminal narrowing SAG: Sagittal.

**Figure 2:**
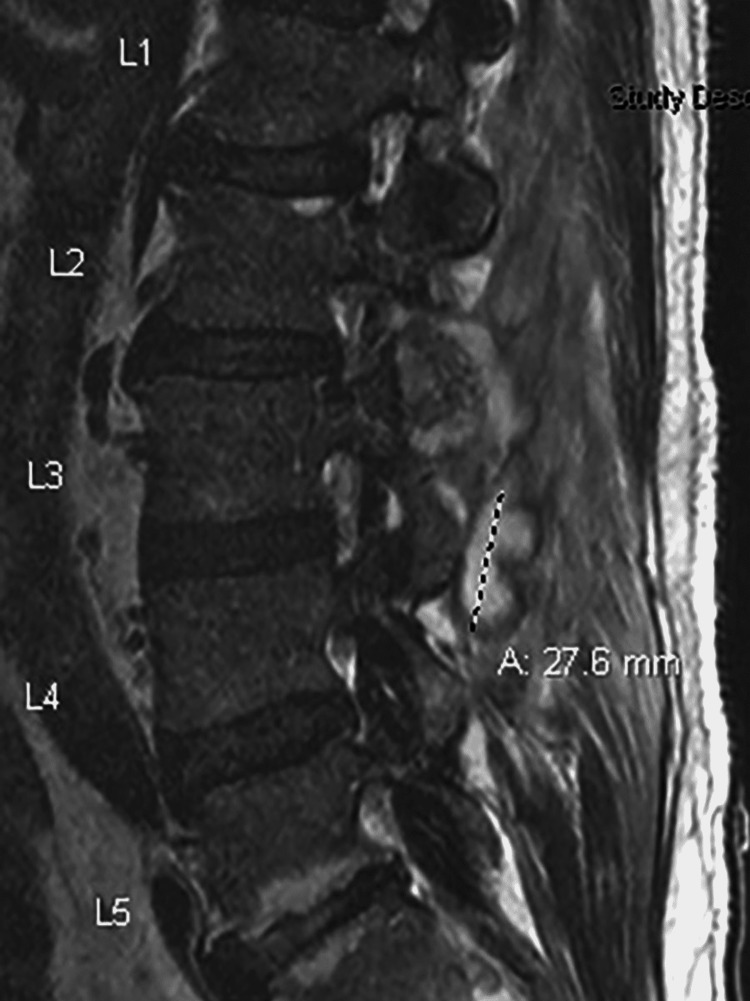
MRI (SAG T2 sequence) showing peripherally enhancing abscess spanning the approximate upper/mid-L3 vertebral body level to the superior L4 vertebral body level, located within the left posterior epidural space of the spinal canal SAG: Sagittal.

**Figure 3:**
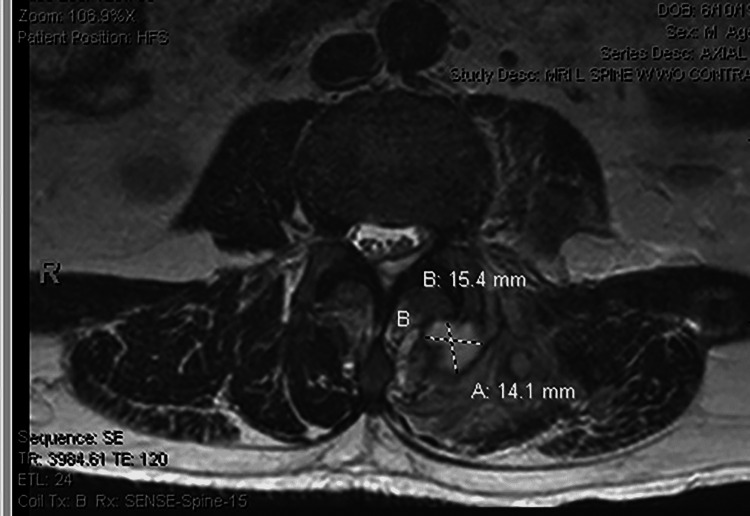
MRI (axial T2 sequence) showing multifocal left paraspinal abscesses and phlegmon, with some possible communication: T2 hyperintense left paraspinal collection at the approximate L4 level measures roughly 1.4 cm (AP) x 1.5 cm (TRV) x 2.8 cm (SI) and abuts the dorsal aspect of the left L3-L4 facet joint. AP: Anteroposterior; TRV: Transverse; SI: Superior to inferior.

Further imaging by MRI of the cervical, thoracic, and lumbar spine showed epidural enhancement from T11 to the sacrum, with large epidural collection from L5 to the sacrum. This was an extensive, long segment of epidural enhancement compatible with epidural phlegmon/abscess, which contributed to varying degrees of moderate/severe foraminal and spinal canal narrowing. The MRI also revealed dural enhancement and leptomeningeal enhancement of the lower thoracic cord extending inferiorly, with a suspected subtle enhancement of the cauda equina nerve roots. Additionally, there was a signal abnormality and enhancement of the left psoas muscle and dorsal paraspinal soft tissues/musculature, most notably on the left, including the presence of edema/inflammatory change and multiple soft tissue rim enhancing collections/abscesses and phlegmon. Abnormal signal and enhancement surrounding the posterior bony vertebral elements were evident to varying degrees. Marrow signal abnormality was likely most conspicuous involving the left posterolateral bony elements of L3, which could be secondary to infection. Lastly, there was a suspicion of a septic facet joint on the left, at L2-L3 and L3-L4. As with x-ray findings, MRI revealed degenerative changes in the lumbar spine (Figures 1-3).

Blood testing included a complete blood count (CBC), showing elevated leukocytes (11.09 K/uL), reduced red blood cell count (3.75 M/uL), low hemoglobin (10.9 g/dL), and low hematocrit (32.8%). A comprehensive metabolic panel (CMP) showed low sodium (132 mmol/L), low chloride (97 mmol/L) and high glucose (283 mg/dL). Erythrocyte sedimentation rate (ESR) inflammatory marker was 116 mm/h. Other tests such as procalcitonin, rheumatoid factor, vitamin B12, lactate, and folic acid were within normal limits. Tick-borne panel and antinuclear antibodies (ANA) were negative. Urinalysis was positive for nitrites and esterase as well as hematuria, proteinuria, and bacteriuria. HbA1c was found to be 13.2%.

Neurology was consulted, given the patient’s symptomology and imaging findings. Physical and occupational therapy was also consulted to assist with the patient’s ambulatory dysfunction and lower back pain with movements. A diabetes educator was consulted to assist with elevated glucose readings.

Differential diagnosis

Amyotrophic Lateral Sclerosis (ALS)

Hallmark features of amyotrophic lateral sclerosis (ALS) classically involve a combination of upper motor neuron (UMN) and lower motor neuron (LMN) signs and symptoms, with asymmetric limb weakness accounting for the most common presentation of ALS. The patient did subjectively endorse lower extremity weakness that was confirmed on physical examination. He also reported limb shaking on standing, which dissipated when walking. Of note, while not present on the examination at the time of admission, the patient did report a history of numbness on the medial aspect of the forearm down to the fourth and fifth digits around a week after the back pain symptoms originally started. On examination, reduced strength was noted in the lower extremities with hyporeflexia of the lower extremities. The timing of his onset of symptoms appeared to be sporadic, and given that the incidence of ALS occurring before the age of 65 is higher in males, neurology was consulted for further evaluation.

Tick-Borne Illness

Northeast Pennsylvania is an endemic region for tick-borne diseases, particularly Lyme disease. Additionally, the patient described living in a wooded area and working outdoors cleaning pond water from toxic run-off. Manifestations during the first days or weeks of infection resemble a non-specific viral syndrome: fatigue, anorexia, headache, neck stiffness, myalgias, arthralgias, regional lymphadenopathy, and fever. Early disseminated disease is characterized by neurologic manifestations, carditis, ocular presentations, and cutaneous findings. Later findings in disseminated disease can present as neurologic manifestations, such as meningitis, cranial neuropathy, and motor or sensory radiculoneuropathy. Neurologic findings in late disease could also manifest differently as a more mild syndrome known as Lyme encephalopathy, characterized by subtle cognitive disturbances. The patient reported new-onset, progressive back pain over a two-month period that was different from his chronic sciatica-type pain. He also reported a total 60-pound weight loss in an eight-month period, with 20 pounds lost over the previous month prior to admission. New-onset expressive aphasia was also reported. The patient was afebrile, and no cutaneous manifestations such as the notable erythema migrans pattern or Bell’s palsy presentation were found on the exam. He did not report feeling excessively fatigued and did not exhibit lymphadenopathy, stiffness, or myalgias. However, given his multiple possible exposures and some suggestive symptoms, lab orders for a tick panel were placed.

Spinal Epidural Abscess (SEA)

Clinical suspicion for SEA occurs in the setting of fever, focal neurologic findings, and new-onset back pain. Suspicion for SEA is heightened in patients with risk factors such as intravenous drug use, chronic indwelling venous catheter, distant infection (i.e., diabetic foot ulcer), older age, or recent spinal manipulation. Though the patient was afebrile, he did have new-onset back pain and focal neurologic deficits presenting as extremity weakness, urinary incontinence, and numbness on the medial aspect of the forearm down to the fourth and fifth digits that began about a week after the back symptoms. In terms of a distant infection, the patient had recurrent episodes of pyelonephritis as well as a pneumonia diagnosis weeks prior to admission. Spinal imaging (x-ray and MRI) was ordered as well and lab workup: CBC, CMP, procalcitonin, ESR, and uric acid (UA).

Treatment

Following MRI confirmation of loculated complex thoracolumbar abscess, the neurosurgery team performed a left L3, L4, L5, and S1 unilateral laminotomy and evacuation of compressive multiloculated epidural abscesses. The patient was then treated with empiric antimicrobial coverage for epidural abscess with vancomycin and ceftriaxone, which was narrowed to cefazolin based on positive MSSA wound cultures obtained in the operating room to complete a total six-week course of antibiotic therapy.

Outcome and follow-up

The patient was discharged five days postoperatively and nine days since the initial presentation on admission. He received home nursing care and completed treatment with intravenous cefazolin via a peripherally inserted central catheter (PICC) line.

The patient was seen by neurosurgery for follow-up approximately two-week postoperatively, one week after hospital discharge. At that time, he reported improvement in back pain, minimal back soreness, and no new leg pain, numbness, or weakness. However, he did have a small portion of proximal surgical incision with superficial wound dehiscence, which later healed without the need for intervention. At a follow-up with the neurosurgery team a few weeks later, the patient was found to have a well-healing surgical site and resolution of the presenting symptoms. Therefore, re-imaging was unwarranted.

Of note, the patient also followed up with his primary care team a few weeks after hospital discharge. He reported improved back pain and was ambulating more around his home. His glucose was better controlled, ranging from 130 to 170 on metformin and subcutaneous basal and short-acting insulin. Lastly, the patient was also seen by infectious disease four weeks post-hospital discharge with a recommendation to complete intravenous cefazolin treatment as planned and to follow up, if necessary.

## Discussion

SEAs are rare, and classic presentations of SEAs are seldom seen. The increasing incidence of SEA in aging populations and in those with chronic diseases makes this diagnosis a crucial one. It can be difficult to consider rare conditions as part of a patient’s differential. However, it might be helpful to be aware of SEA risk factors and link them to the onset of emerging symptoms, particularly if it includes the classic triad of fever, back pain, and neurological deterioration.

However, SEA may also present with more benign variations. Clinical suspicion should be high in individuals with intermittent back pain, deteriorating neurological function, a history of treated infections rather than fever, and immunocompromised conditions such as uncontrolled diabetes or other causes of a compromised immune system such as chronic glucocorticoid use [6].

In the case of this patient, his past medical history and social history complicated the differential and encouraged our team to think broadly. He had a history of chronic, ongoing pain due to multiple past orthopedic surgeries involving his legs and back as well as long-standing sciatic pain. Initially, the treatment team's differential included mechanical causes, neurodegenerative diseases, possible toxic exposures, as well as infectious sources. In the end, the acute onset of progressive back pain that the patient described was different from his sciatica pain, thus leading to higher prioritization of SEA as the cause and imaging of the spine to further investigate these changes.

While it is thought that 20% of SEA cases are idiopathic, the remaining are likely from bacterial sources [7]. In 50% of cases, bacteria from the skin, soft tissue, urinary, or respiratory system infiltrate the epidural space hematogenously. In 10%-30% of cases, the infection comes from adjacent infections such as vertebral osteomyelitis or psoas myositis. The remaining 15% of cases are brought on by invasive procedures such as lumbar punctures, epidural injections, and other neurosurgical interventions [6]. Regarding the identification of a source of infection, the patient had recurrent episodes of urinary tract infections and pyelonephritis leading to his admission to the hospital. With this type of chronic infection, the source could have come from the pelvis and the venous system, thereby infecting the spinal cord [8,9]. Another possibility could be his untreated diabetes, which has been reported as a high-risk factor for developing SEA [4,5]. Lastly, another source of infection could have been the psoas and dorsal paraspinal myositis noted on imaging. In the end, it is difficult to say for sure which was the precipitating factor of this SEA.

Aside from the need to diagnose SEA as soon as possible, the challenge is to coordinate the care of these cases with interprofessional teams of physicians, nurses, and physical therapists. These teams, in turn, help manage a multitude of potential complications associated with SEA and post-treatment recovery, including pressure ulcers, deep vein thrombosis (DVT), sepsis, urinary retention, supine hypertension, gastric peristalsis, constipation, and muscle weakness [5]. Though surgical treatment may provide immediate improvements, SEA cases generally recover over time and may require rehabilitation to regain function, depending on the extent of the abscess and time to diagnosis. In the case of this patient, he had significant improvement immediately after the surgical evacuation of his SEA. Over the course of several months, he continued to improve and regain his strength, but he had not yet returned to work. He is continuing to recover and is being monitored by his medical care team.

This case demonstrates the importance of timely diagnosis and treatment for patient safety, the immediate aim of surgical intervention to evacuate the abscess, and the long-term goal of preventing potentially life-threatening, irreversible complications or disease sequelae. The most challenging part of this case was keeping SEA as a differential when it did not present classically. However, the benefits of keeping a rare condition such as SEA within the differential far outweigh any costs associated with imaging and other testing. This is particularly true when the time to diagnosis can mean the difference between the restoration of function through treatment and recovery versus morbidity or mortality.

## Conclusions

Classic presentations of SEA are rare. However, a high index of suspicion and knowledge of SEA risk factors are required to make the diagnosis promptly to avoid irreversible neurologic or even fatal outcomes. Diabetes is associated with 15%-33% of multiple SEA case series. Other chronic diseases, such as alcoholism, end-stage renal disease (ESRD), HIV, organ transplantation, indwelling catheters, IV drug use, and systemic infections, that impair immunity are also associated with SEA.
